# Effects of parathyroidectomy on tumoral calcinosis in uremic patients with secondary hyperparathyroidism

**DOI:** 10.1186/s12893-019-0603-8

**Published:** 2019-09-11

**Authors:** Jing Wang, Ming Zeng, Guang Yang, Yaoyu Huang, Buyun Wu, Jing Guo, Ningning Wang, Changying Xing

**Affiliations:** Department of Nephrology, The First Affiliated Hospital of Nanjing Medical University, Nanjing, 210029 Jiangsu Province China

**Keywords:** Tumoral calcinosis, Secondary hyperparathyroidism, Parathyroidectomy, Phosphate

## Abstract

**Background:**

Tumoral calcinosis (TC) is a rare disease derived from uremic secondary hyperparathyroidism (SHPT). However, parathyroidectomy (PTX) seems to be ineffective at relieving TC in some patients. In this study, we investigated the relationship between PTX and TC shrinkage.

**Methods:**

We retrospectively followed up nine TC patients who underwent PTX, dividing them into two groups: those with TC size reduced by > 80% were in the “effective group” (group A), and the rest in the “ineffective group” (group B).

**Results:**

We enrolled nine patients (7 men; mean age 38.6 ± 10.9 years) with SHPT-related TC. One patient with calciphylaxis was excluded due to sudden death. The efficiency of PTX in causing TC regression was 62.5% (5 patients in group A). Group A had a shorter overall duration of TC (6 [5.5, 6.0] vs. 9 [8.0, 10.0] months; *P* = 0.02) and higher serum levels of alkaline phosphatase (ALP; 408.0 [217.9, 1101.7] vs. 90.8 [71.0, 102.1] pg/ml; *P* = 0.03) and high-sensitivity C-reactive protein (hs-CRP; 82.7 [55.0, 112.4] vs. 3.1 [3.1, 4.5] mg/l; *P* = 0.02). Average calcium supplementation within 1 week of surgery was significantly greater in group A than in group B (96.8 [64.1, 105.3] vs. 20.1 [13.1, 32.7] g; *P* = 0.04). Patients in both the groups demonstrated similar serum phosphate levels before PTX, but these levels were higher in group B than in group A at follow-up times (3 months, *P* = 0.03; 6 months, *P* = 0.03).

**Conclusions:**

The shorter duration of pre-existing TC and higher ALP levels before PTX, as well as lower serum phosphate levels after PTX, were correlated with effective SHPT-TC shrinkage.

## Background

Tumoral calcinosis (TC) is an uncommon end-stage renal disease (ESRD)-related complication in bone and mineral metabolism, with calcium phosphate deposits occurring in soft tissues. These deposits predominantly form around large articular areas and cause intolerable pain and skin ulceration [1, 2]. Epidemiological data indicate that morbidity is approximately 0.5–3% in patients receiving hemodialysis. TC might originate from secondary hyperparathyroidism (SHPT). The main reasons explaining this finding include high-turnover bone disease, treatment of hyperphosphatemia with calcium-containing phosphate binders and active vitamin D, and increased calcium and phosphate loads [3]. These conditions result in increased levels of serum calcium, phosphate and calcium × phosphate product, eventually leading to extraosseous calcification [4–6]. Previously, only sporadic cases of TC have been noted [7–9]. In our clinical practice, we encountered a small cohort of SHPT patients with TC (SHPT-TC) combined with slight or overt articular-movement disorder. Clinical manifestations, laboratory results and pathological properties in such populations need to be characterized.

Kidney transplantation or parathyroidectomy (PTX) seems to be an effective treatment for alleviating SHPT-TC and improving patients’ quality of life [9–11]. However, hormone deprivation therapy based on successful PTX did not succeed in shrinking TC in some patients [8]. The aim of this study was to investigate the relationship between PTX and TC shrinkage.

## Methods

### Patient population

From August 2012 to December 2017, SHPT was diagnosed in 597 patients, and of these, we retrospectively identified nine SHPT-TC patients (7 men; mean age 38.6 ± 10.9 years). All patients underwent total PTX with forearm auto-transplantation. We recorded the patients’ genders, ages, durations of dialysis and medical histories. Patients were divided into two groups: Group A, the “effective group,” was defined as having TC size reduced > 80%; we placed all other patients in Group B, the “ineffective group.” This study was approved by the Human Research Ethics Committee of the First Affiliated Hospital of Nanjing Medical University, Nanjing, China. All patients provided written informed consent.

### Definitions of successful PTX

According to previous studies, serum intact parathyroid hormone (iPTH) levels detected at the first postoperative week < 300 pg/mL were the criterion for successful PTX [12].

### Biochemical examination

Testing included routine blood and complete blood counts (CBCs), high-sensitivity C-reactive protein (hs-CRP), iPTH, 1,25 vitamin D, hemoglobin, glucose, creatinine, urea, albumin, calcium, phosphorus and alkaline phosphatase (ALP).

### Imaging and pathological examination of TC

Each patient underwent comprehensive imaging, including plain radiography, local computed-tomography (CT) scans with three-dimensional reconstructions and (99 m)Tc-Methyl diphosphonate [(99 m)Tc-MDP] bone scintigraphy. We biopsied masses from five patients. For histological analysis, specimens were processed in formalin and embedded in paraffin; we performed hematoxylin and eosin (H&E) staining. Cell-counting analyses were carried out by an observer blinded to the analysis of tissues on images acquired at × 20 magnification. The number of multinuclear giant cells (MNGCs) over an area of 0.25cm^2^ was expressed as cells per unit area.

### Post-PTX follow-up

After PTX, we assessed serum calcium, phosphate and iPTH levels on day 7 and at 3 and 6 months. Hungry-bone syndrome (HBS) was defined as hypocalcemia with corrected serum calcium level < 2.0 mmol/L after PTX [13]. We performed plain radiography or CT scanning during follow-up.

### Statistical analysis

We performed all statistical analyses using the Statistical Package for the Social Sciences (SPSS) version 20.0 (SPSS Inc., Chicago, IL, USA). Continuous variables were presented as mean ± standard deviation (SD) or quartiles, and categorical variables were presented as numbers and proportions. We compared differences between groups using a nonparametric test for continuous variables and Fisher’s exact test for categorical variables. *P* < 0.05 was considered statistically significant.

## Results

### Patient characteristics

We enrolled a total of nine patients (7 men; mean age 38.6 ± 10.9 years) with SHPT-TC (see Table 1). One patient who developed calciphylaxis (case 7; see Table 2) suddenly died within 1 month of surgery and was excluded. All patients had intermittently taken phosphate binders of calcium carbonate and calcitriol pulse therapy for > 3 months. In this study, after PTX, TC resolved in 5 patients (Group A) and did not resolve in 3 patients (Group B). The clinical characteristics between the 2 groups are summarized in Table 1. Group A had higher iPTH levels (2532.8 [1134.0, 2934.9] vs. 629.0 [422.2, 1300.0] pg/ml; *P* = 0.10), although there was no statistical difference between the groups. Group A had shorter durations of TC (6.0 [5.5, 6.0] vs. 9.0 [8.0, 10.0] months; *P* = 0.02) and increased serum levels of ALP (408.0 [217.9, 1101.7] vs. 90.8 [71.0, 102.1] pg/ml; *P* = 0.03) and hs-CRP (82.7 [55.0, 112.4] vs. 3.1 [3.1, 4.5] mg/l; *P* = 0.02). All patients were supplemented with calcium and vitamin D due to immediate development of hypocalcemia (< 2.0 mmol/L) after PTX.

**Table 1:** Comparison of clinical characteristics between effective and ineffective groups

Characteristic	Effective group (Group A)	Ineffective group (Group B)	*P* value
No. of patients (n)	5	3	–
Age, years	39.8 ± 14.5	37.0 ± 7.0	0.72
Male, n (%)	4 (80)	2 (67)	0.64
Course of TC, months	6 (5.5, 6.0)	9 (8, 10)	0.017
Serum iPTH, pg/ml	2532.8 (1134.0, 2934.9)	629.0 (422.2, 1300.0)	0.101
Serum Calcium, mmol/l	2.3 (2.3, 2.5)	2.22 (2.21, 2.35)	0.26
Serum Phosphate, mmol/l	2.6 (2.4, 2.8)	2.31 (2.18, 2.84)	0.57
Serum Magnesium, mmol/l	1.02 (0.86, 1.26)	1.11 (0.76, 1.13)	0.655
Serum 25-hydroxyVitamin D, ng/ml	62.9 (34.9, 77.5)	96.6 (29.2, 97)	0.297
Serum alkaline phosphatase, U/L	408.0 (217.9, 1101.7)	90.8 (71.0, 102.1)	0.025
hsCRP, mg/l	82.7 (55.0, 112.4)	3.11 (3.11,4.5)	0.024

Abbreviation: *TC* tumoral calcinosis, *iPTH* intact parathyroid hormone, *hsCRP* high-sensitive C-reactive protein

**Table 2:** Clinical features and time of absorption after PTX

NO	Group	TC duration	Time of absorption	Location	Operation method	Follow up
4	A	6 months	1 months	RE RK LS	PTX	11 months
5	A	6 months	4 months	RS ST	PTX	41 months
6	A	5 months	3 months	LF RS	PTX	19 months
8	A	6 months	3 months	LB	PTX	28 months
9	A	6 months	2 months	RB	PTX	45 months
1	B	8 months	unabsorbed	RB	PTX	14 months
2	B	9 months	unabsorbed	LB RS RT	PTX + surgical excision	29 months
3	B	10 months	unabsorbed	RB	PTX	15 months

Abbreviation: *PTX* parathyroidectomy, *RE* right elbow, *RK* right knee, *LS* left shoulder, *RS* right shoulder, *ST* the sternum, *LB* left buttock, *LF* Left forearm, *RB* right buttock, *LB* left buttock, *RS* right shoulder, *RT* right thigh

### Clinical properties and regression of TC after PTX

**Fig. 1:**
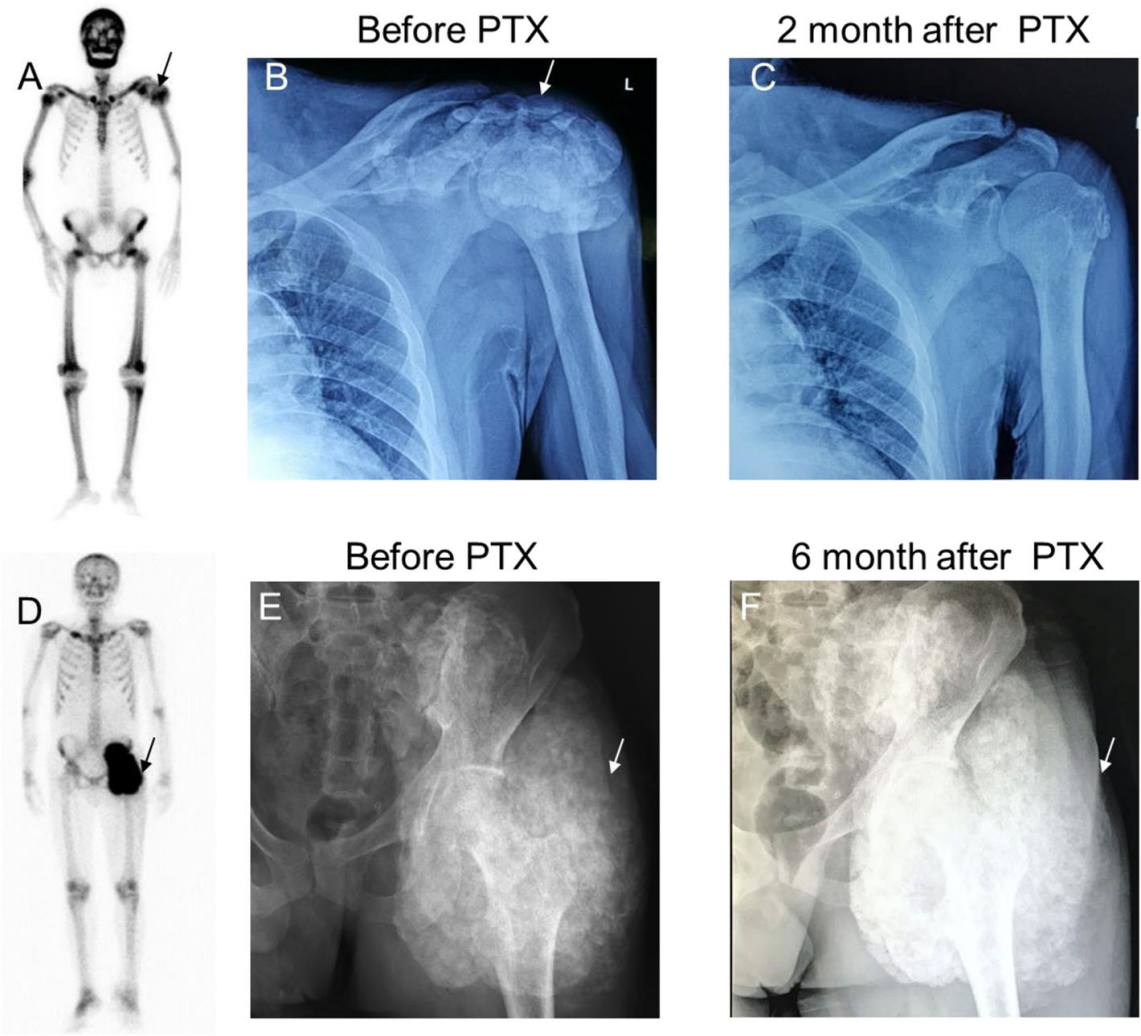
Examples of TC regression in two groups. **a**, **b** TC was detected by bone scintigraphy and plain radiographs in case 5. **c** TC was absorbed 2 months after PTX. **d**, **e** TC was detected by bone scintigraphy and plain radiography in case 1. **f** TC was not relieved 6 months after PTX. Arrows indicate TC location. TC, tumoral calcinosis

Clinical profiles of patients with TC are summarized in Table 2. Physical examination revealed calcium phosphate deposits predominantly formed around large articular areas. These led to moderate-to-severe limitation of passive and active ranges of motion in all patients, including 1 who suffered intolerable pain and upper-limb numbness due to ulnar-nerve compression. All symptoms were relieved in group A after PTX (Fig. 1a–c). Absorbed cases exhibited postoperative clinical regression; that is, softening of swelling; rapid reduction in size and rapid remission of masses within 4 weeks; and disappearance of masses within 4 months. In group B, as shown in Fig. 1d–f, we detected TC by bone scintigraphy and in case 1, plain radiography (see Table 1) before PTX; the mass was unabsorbed at 6 months after PTX.

### Dynamic monitoring of serum iPTH, calcium and phosphate levels after PTX

All patients had successful PTX. As shown in Table 3, serum iPTH levels on day 7 and at 3 and 6 months post-operation did not significantly differ between the two groups. In addition, we observed similar trends in serum calcium levels. Serum phosphate levels in group B were higher than those in group A at 3 and 6 months’ follow-up (3 months, 1.2 [0.7, 1.7] vs. 2.1 [2.0, 2.2] mmol/l; 6 months, 1.4 [0.9, 1.8] vs. 2.4 [2.3, 3.0] mmol/L). Total calcium supplementation within 1 week of surgery was significantly greater in group A than in group B (96.8 [64.1, 105.3] vs. 20.1 [13.1, 32.7] g; *P* = 0.04).

**Table 3:** Dynamic monitoring of serum iPTH, Ca and P levels after PTX procedure

Follow-up	Effective group (Group A)	Ineffective group (Group B)	*P* value
Serum iPTH, pg/ml	2532.8 (1134.0, 2934.9)	629.0 (422.2, 1300.0)	0.101
7 days	21.5 (7.7, 190.4)	56.0 (43.4, 70.9)	0.79
3 months	72.0 (50.4, 126.1)	86.0 (65.0, 130.4)	1.00
6 months	82.7 (58.9, 130.7)	56.5 (45.7, 78.0)	0.25
Serum Calcium, mmol/l	2.3 (2.3, 2.50)	2.2 (2.2, 2.4)	0.26
7 days	2.2 (2.1, 2.3)	2.0 (1.9, 2.5)	0.65
3 months	2.0 (1.8, 2.2)	2.3 (2.1, 2.4)	0.25
6 months	2.0 (1.9, 2.2)	2.1 (2.0, 2.5)	0.393
Average dosage of calcium supplement ^a^			
7 days (g)	13.8 (9.2, 15.0)	2.9 (1.9, 4.7)	0.036
Serum Phosphate, mmol/l	2.6 (2.4, 2.8)	2.3 (2.2, 2.8)	0.57
7 days	1.1 (0.9, 1.4)	1.6 (1.4, 1.8)	0.053
3 months	1.2 (0.7, 1.7)	2.1 (2.0, 2.2)	0.025
6 months	1.4 (0.9, 1.8)	2.4 (2.3, 3.0)	0.025

Abbreviation: *iPTH* intact parathyroid hormone, *Ca* Serum Calcium, *P* Serum Phosphate, *PTX* parathyroidectomy

^a^:Cumulative dose of calcium supplement within 1 week postoperatively, in the form of oral calcium carbonate tablets (40% elemental calcium) and intravenous calcium gluconate (9% elemental calcium)

### Histopathology properties of TC

**Fig. 2:**
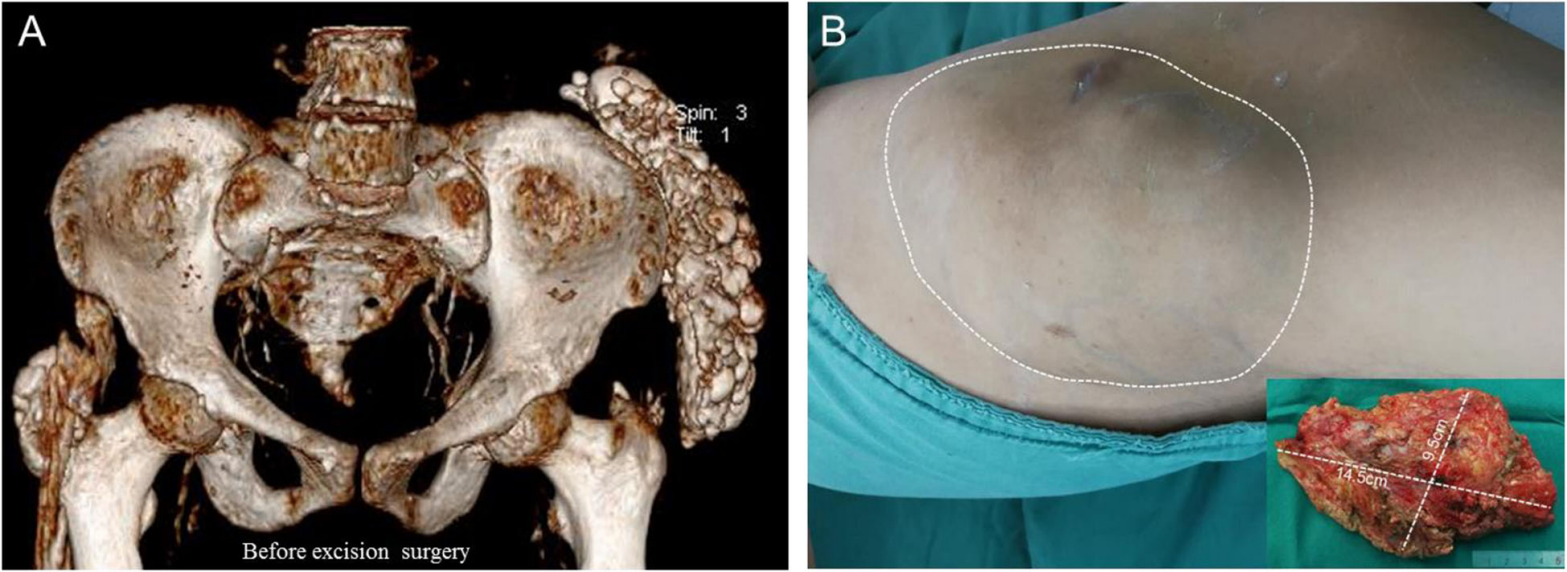
Surgical excision of mass in case 2. **a** CT scans with 3D reconstructions showed remarkable ectopic calcifications in the left buttock that limited left-hip mobility. **b** The size of the mass resected from the left buttock was 14.5 × 9.5 cm^2^

**Fig. 3:**
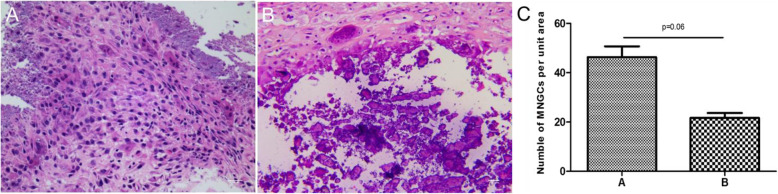
Histopathological properties of TC in both groups. **a** and **b** H&E staining revealed chronic inflammation and multiple calcified granulation tissues. MNGCs were visible at the edge of the calcified tissue. **c** There were more MNGCs in group A than in group B (*P* = 0.06)

Six patients (3 in group A) underwent mass biopsies before PTX. In group B, 2 patients refused local surgical resection, and at 18 months’ follow-up a mass was excised from the left buttock in case 2 (see Table 2). The size of the resected mass was 14.5 × 9.5 cm^2^ (Fig. 2). During excisions biopsies, the calcified masses had numerous septated cysts containing milky fluid and granular basophilic material. H&E staining revealed chronic inflammation with foam macrophages and multiple calcified granulation tissues. Adjacent to the border of the calcified tissue, MNGCs were visible with H&E staining (Fig. 3). Compared with group B, group A had more MNGCs (46.3 ± 7.6 vs. 21.7 ± 3.5; *P* = 0.06).

### Complications

No complications were observed in the study during the perioperative PTX period.

## Discussion

TC is a rare complication characterized by progressive growth of painful calcium phosphate deposits within juxta-articular areas [1]. Based on the pathogenetic classification, TC is divided into three subtypes: (1) primary normophosphatemic TC, (2) primary hyperphosphatemic TC and (3) secondary TC. Secondary TC might originate from chronic kidney disease (CKD)-related SHPT, bone neoplasm or hypervitaminosis D [14–16]. The complication occurs 8–36 months after the onset of SHPT, and the morbidity is 0.5–3.0% [4, 5, 17, 18]. We observed a similar finding in our study (1.5% morbidity rate; 9 of 597 patients). According to previous studies [19, 20], when patients were refractory to treatment by internal medicine, they underwent total PTX with forearm subcutaneous auto-transplantation.

There are several treatment strategies for TC. Conservative medical management involves restricting calcium-based phosphate binders in hyperphosphatemic patients across all severities of CKD [12], decreasing dialysis calcium concentration and increasing hemodialysis intensity [21]. Another treatment choice for soft-tissue calcification is ultrasound-guided aspiration with anesthetic and steroid injection [22]. Surgical resection of masses can be performed as a last resort [9, 17]. According to rare case reports, PTX is considered an effective treatment for TC in hemodialysis patients with SHPT [7, 9]. However, hormone deprivation therapy based on successful PTX did not succeed in shrinking TC in some patients [8]. The reasons for these different clinical regressions have not been demonstrated. In our study, PTX’s efficiency in causing TC regression was 62.5%. Compared with the ineffective group at baseline, the effective-regression group had shorter durations of TC (*P* < 0.02) and higher serum levels of ALP (*P* = 0.05) and hs-CRP (*P* = 0.01). Serum phosphate levels in group B were higher than those in group A at follow-up times (3 and 6 months; *P* < 0.05).

Reasons for TC absorption in our study might be as follows: (1) higher serum levels of ALP before PTX. HBS commonly develop after PTX, characterized by increased bone formation in parallel with cessation of bone resorption as well as extraosseous calcification dissolution [23–26]. All patients in our study developed HBS postoperatively. Increased pre-operative serum ALP levels were predictive of prolonged HBS duration following surgery [23]. Furthermore, average calcium supplementation within 1 week of surgery was significantly greater in group A, which had higher baseline ALP levels than group B did. Occurrence of hypocalcemia was correlated with a rapid decrease in serum iPTH level, resulting in a shift of calcium influx to the skeletal system [23, 27, 28]. (2) Shorter duration of TC. Longer treatment duration might lead to formation of calcium hydroxyapatite crystals surrounded by connective tissue composed of fibroblasts and compressed collagen fibers, which is hardly absorbed. This hypothesis was borne out by histological evaluation in our study and in previous studies [8, 15]. (3) Increased pre-operative serum hs-CRP levels and reduced postoperative serum phosphate levels. It has been recently demonstrated that macrophages are associated with mineralization absorption and can transdifferentiate into osteoclastic cells and degrade calcified elastin [29, 30]. In our study, the higher the levels of hs-CRP before PTX, the more MNGCs (osteoclastic cells) we identified around the border of the calcified tissue. Furthermore, Mozar’s study suggests that a high extracellular inorganic-phosphate concentration inhibits bone-resorbing activity in osteoclast-like cells [31]. We observed lower phosphate levels after PTX in group A than in group B.

Clinically, TCs were dissolved within 1–5 months in accordance with previous case reports [7, 9] and our study. Surgical excision is suggested if SHPT-TCs are not absorbed by the 6-month follow-up. In patients with TC due to SHPT, earlier PTX is suggested to be an effective strategy for the treatment of TC.

### Study limitations

First, this study involved a small number of patients due to the rarity of tumoral calcinosis in uremic secondary hyperparathyroidism patients, and the follow-up time is short. Second, we did not dynamically detect circulating inflammation, bone reabsorption or formation markers to evaluate the effects of PTX on systemic inflammatory and bone metabolism disorders. Third, no experiments were performed to verify the above mechanisms.

### Clinical implications

In patients with tumoral calcinosis due to secondary hyperparathyroidism, earlier PTX is suggested to be an effective strategy for the treatment of TC.

## Conclusions

The shorter duration of pre-existing TC, higher serum pre-operative ALP levels and lower postoperative phosphate levels were correlated with effective SHPT-TC shrinkage.

## Data Availability

Not applicable. We consulted to all of the patients, they refused to upload the raw data.

## Abbreviations

(99 m)Tc-MDP: (99 m)Tc-methylene diphosphonate
ALP: Alkaline phosphatase
CBC: Complete blood counts
ESRD: End-stage renal disease
HBS: Hungry bone syndrome
HE: Hematoxylin and eosin
hs-CRP: High-sensitivity C-reactive protein
iPTH: Intact parathyroid hormone
PTX: Parathyroidectomy
SHPT: Secondary hyperparathyroidism
SHPT-TC: SHPT patients with TC
TC: Tumoral calcinosis
